# Telemedicine Experience in China: Our Response to the Pandemic and Current Challenges

**DOI:** 10.3389/fpubh.2020.549669

**Published:** 2020-12-02

**Authors:** Yuye Wang, Baojie Li, Lei Liu

**Affiliations:** ^1^Department of Neurology, The First Affiliated Hospital of China Medical University, Shenyang, China; ^2^Department of Public Service, The First Affiliated Hospital of China Medical University, Shenyang, China

**Keywords:** COVID-19, telemedicine, remote consultation, public health event, countermeasures and suggestions

## Introduction

Coronavirus disease 2019 (COVID-19), a sudden public health event, has attracted the attention of the whole society. In recent months, medical workers around the world have been fighting against covid-19. How to use modern scientific and technological methods to maximize the therapeutic effects and prevent diseases has become the focus of attention. Telemedicine, which combines enhanced hardware, digital medicine, image capture and disease diagnosis, can eliminate the limitation of distance and provide high-quality expert services. Previous work has described the application of telemedicine in disasters (1) and public health emergencies (2). However, after the outbreak of COVID-19, the process of medical resource allocation becomes more difficult, and we need new management methods to maintain a balanced allocation of resources. Therefore, telemedicine tends to be a good strategy to provide care for COVID-19 patients.

## What We Did?

Specialist care disparities exist worldwide and were especially evident during the epidemic period. Under such circumstances, experts took advantage of telemedicine to connect with the medical teams supporting Hubei province, providing remote consultation and case discussion for critically ill patients. Telemedicine service no doubt enhanced the ability and increased the cure rate especial for severe cases (no death occurred in all cases under telemedicine) in the local area. From 1st February to 31th March, 358 patients with COVID-19 received emergency tele-consultations (*n* = 25) or tele-image (*n* = 333) and become better after taking expert instructions. In addition to connection with Hubei, experts also connected with treatment centers in less developed areas (such as Kuandian County in Dandong City and Fuxin City) in order to establish an expert team and complete multidisciplinary treatment of critically ill patients. The consultation involved more than 10 departments including intensive care unit, department of respiratory, radiology, neurology, endocrinology, gastroenterology, and so on, which effectively makes the best use of the comprehensive medical resources. Experts gathered in telemedicine center or used a real-time, synchronous videoconferencing to provide real-time assistance. Compared with the treatment of critically ill patients, the diagnosis and exclusion of suspected patients in primary hospitals is more difficult, which often requires the support of expert groups. In order to save time and serve more patients (out of hospital), we used a remote consultation platform to connect experts (in Third-level grade hospital) with frontline health workers (in community healthcare center) and shared medical images, record data, and test results. Moreover, this approach can potentially avoid or reduce the need for face-to-face consultations, protect experts from exposure to viruses and save costs.

Additionally, the telemedicine center set up a platform for experts to carry out interpretation of the guidelines for the diagnosis and treatment of COVID-19 and methods for nosocomial infection control, which improved the ability of medical workers to fight against the Severe acute respiratory syndrome coronavirus 2 (SARS-CoV-2) and self-protection. Medical workers could share the opinions and experiences with each other in order to achieve more suitable treatment methods. Psychologists provided psychological counseling for frontline doctors through videoconferencing to help them cope with stress and anxiety. In parallel, experts gave lectures on how to protect susceptible population which would reduce public panic about the epidemic. Besides, from 20st February to 31st March, medical workers answered the questions from more than 3,000 patients and suspected population through live video which saves the repeated workload and time of ward rounds and reassure the patients' emotions. Others could achieve the information they want through playback.

In order to avoid crowd gathering and cross-infection, many patients without COVID-19 infection could not go to medical institutions for treatment. Due to lack of protective materials, the order of ordinary clinics was disrupted. Under such background, the remote platform (mobile phone to mobile phone) conducted consultations and guidance for patients with other diseases (~2,400) and carried out online medical activities. We also had an online outpatient service to offer treatment methods for people with chronic disease, mental illness or other diseases, which is convenient for patients to get treatment. Moreover, online services could prevent patients with mild symptoms from taking up high-quality medical resources, increase the availability of doctors from less developed medical institutions as well as save time.

## Future Requirements

With the development of information communications technology (ICT), many medical institutions have established their own remote platforms. However, it is particularly important to break the barriers between different remote platforms and achieve rapid connection when sudden public events happen. In the future, we need to develop new technologies and explore new mechanisms to ensure the rapid connection among different remote platforms in emergency situations.

Besides, there are inconsistency between the imaging platforms and medical record management systems, which increases the difficulty for experts to get relevant case information. Nowadays, the initiator needs to use a USB flash disc to send the images and medical records information to the receiver according to the telemedicine system. In the future, we need to explore new approaches to ensure the rapid information transmission among different remote platforms.

In order to achieve real time response, the telemedicine center should have personnel on duty 24 h a day and expert groups are informed to be well-prepared for emergency severe case consultation at any time. At present, we are developing and exploring more scientific response methods and mechanisms, such as reminders integrated with remote consultation systems, intelligent telemedicine service automatic receiving systems and so on, to improve work efficiency and save labor costs.

Investment should be increased so that telemedicine can cover more hospitals. Because the construction and maintenance of the telemedicine service system and the technical training of workers need financial support. In less developed areas, the lack of high-speed broadband access may account for the inability to reach underserved populations. However, with the promotion and implementation of 5G, we believe that telemedicine would serve more residents in the future. We support the idea that telehealth should be fully utilized not only in emergencies, but also in daily routine care (3, 4).

Besides, telemedicine providers must be credentialed and must have a license to practice medicine which should be under supervision of relevant departments. Online training sessions should be provided for clinicians and patients who require just-in-time training or assistance on their first call. In addition, whether or not the costs incurred are covered by health insurance, ethical problems and network security issues in remote transmission should also be considered at the same time.

To sum up, major requirements include (1) unifying remote platform; (2) developing a real time response mechanism; (3) increasing investment; (4) training professional specialist; (5) medical insurance covering. With the resolution of these problems, the utility of telemedicine in public health emergency and routine care will be greatly improved, which also provides a reliable guarantee for the response and disposal of medical ICT in China.

## Discussion

In developed countries, telemedicine has been widely used in chronic disease management (5). As a special patient group, the rapid application of telehealth in obstetrics and gynecology (6) and mental health monitor and control (7) during the pandemic has also been proposed. A forward triage of “direct-to-consumer telemedicine” is proposed to effectively screen patients (8), where only medium to high-risk patients are advised to go to fever clinic. Further promotion of this scheme in China will allow patients to be monitored at home, which also saves the workload of triage in hospital and avoids exposure of nurses. In western China, during the pandemic, a multimodal telemedicine network synergizing a 5G service, a smartphone application, and telemedicine system was also established in Sichuan Province (9) which is similar to us. However, most patients are comfortable with in person or face to face consultations with their specialist. People with insufficient cognition or physical impairment may find it difficult to interact with a telemedicine platform, which may result in the low application rate of telemedicine.

With the development of ICT, the 5G era has arrived. The application of telemedicine is not limited to consultation, and the 5G technology is also used in remote robotic surgery and remote ultrasound examination. Due to the improvement of artificial intelligence, robots are used to deliver medicines in the ward. It is believed that in the near future, remote ventilator regulation, intelligent ward inspection and handling systems will be implemented, where the smartphone applications could assess respiratory rate and other physical indicators. Therefore, the possibility of infecting medical personnel during the epidemic can be largely avoided.

Recently, synchronous network broadcast has been widely used. In February, the construction of the HuoShenShan and LeiShenShan hospital was shown to all people concerned about Wuhan through live video, which undoubtedly inspired the Chinese people. According to recent reports, Chinese experts also share their experience in fighting against COVID-19 with experts from other countries via video conferencing (10, 11). President Xi connected with infectious ward in HuoShenShan hospital through video call to show his concern about the patients and gratitude to medical workers.

In conclusion, during the epidemic, telemedicine not only enabled remote consultation, but also simultaneous image transmission and information communication, which greatly reduced the pressure on frontline medical staff. The world is facing a critical situation with increasing cases of covid-19. To date, China has been actively fighting against the COVID-19 and has achieved positive results. Using ICT to establish a real-time, efficient, and convenient telemedicine platform will comprehensively improve our ability to deal with public health emergencies, which will also improve the diagnosis and treatment of critical ill patients, coordinate and optimize resource allocation, and avoid the spread of diseases caused by the transfer of patients. Telemedicine has the potential to provide patients with more regular and efficient care than traditional models of care. A more effective telemedicine system should be established by promoting such medical services. Analyzing problems encountered in the practical applications is also conducive to the sustainable development of the telemedicine system and can provide an important reference for dealing with public health emergencies and routine care in the future. We hope to share our experience with the world and help other developing countries fight against the disease pandemic.

## Author Contributions

LL and BL contributed to the conception of the opinion. YW wrote the manuscript. All authors contributed to the article and approved the submitted version.

## Conflict of Interest

The authors declare that the research was conducted in the absence of any commercial or financial relationships that could be construed as a potential conflict of interest.
